# Effects of a Virtual Reality–Based Natural Environment Intervention on Attention and Mood in Community-Dwelling Older Adults: Randomized Controlled Trial

**DOI:** 10.2196/87861

**Published:** 2026-02-25

**Authors:** Cheng-Chen Chou, Jong-Ni Lin, Chia-Pin Yu, Chi-Wen Chen, An-Yun Yeh, Yen-Kuang Lin

**Affiliations:** 1 Institute of Community Health Care College of Nursing National Yang Ming Chiao Tung University Taipei Taiwan; 2 Master Program in Transdisciplinary Long-Term Care and Management National Yang Ming Chiao Tung University Taipei Taiwan; 3 Department of Post-Baccalaureate Nursing/ Department of Nursing College of Nursing and Health Sciences Da-Yeh University Changhua Taiwan; 4 School of Forestry and Resource Conservation National Taiwan University Taipei Taiwan; 5 Department of Nursing College of Nursing National Yang Ming Chiao Tung University Taipei Taiwan; 6 Department of Nursing College of Staten Island The City University of New York Staten Island, NY United States; 7 Graduate Institute of Athletics and Coaching Science National Taiwan Sport University Taoyuan Taiwan

**Keywords:** virtual reality, natural environment, community-dwelling older adults, attention, mood

## Abstract

**Background:**

Aging is a phenomenon accompanied by declines in attention and emotional well-being. Exposure to natural environments has been shown to support cognitive and psychological restoration; however, many older adults face barriers to accessing authentic natural environments. Virtual natural environments may offer an accessible alternative, but evidence from community-dwelling older adults remains limited.

**Objective:**

This study examined the immediate effects of a single-session virtual or actual-nature exposure on attention, mood, and physiological indicators of stress among community-dwelling older adults compared with actual-nature and urban environments.

**Methods:**

A randomized controlled trial with a 3-group, pre-post design was conducted among 120 community-dwelling adults aged 65 years and older in northern Taiwan. Participants were randomly assigned to 1 of 3 groups: a virtual-nature group, an actual-nature group, or an actual-urban environment group. Then, they completed a single 20-minute exposure session. Attentional performance, mood, and physiological indicators were assessed before and immediately after the intervention. Intervention effects were analyzed using generalized estimating equations, focusing on group-by-time interaction effects.

**Results:**

Compared with the urban environment group, the virtual-nature group demonstrated a significantly greater improvement in attentional capacity, as measured by the digit span test (B=1.00, 95% CI 0.19-1.80; *P*=.02), and significant improvements in both positive affect (B=2.82, 95% CI 0.02-5.62; *P*=.048) and negative affect (B=–2.22, 95% CI –4.05 to –0.39; *P*=.02). The actual-nature group showed a significant improvement in positive affect compared with the urban group (B=3.92, 95% CI 1.65-6.19; *P*=.001) but did not demonstrate a significant improvement in attention. No significant group-by-time interaction effects were observed for physiological indicators. No adverse events were reported.

**Conclusions:**

A single 20-minute virtual-nature exposure was associated with immediate improvements in attention and mood among community-dwelling older adults, whereas physiological effects were not detected. These findings suggest that virtual nature may serve as an accessible, short-term restorative strategy, particularly when access to real nature is limited. Larger, multisession trials with longer follow-ups are needed to evaluate the sustained effects of virtual-nature interventions for mental well-being in older populations.

**Trial Registration:**

ClinicalTrials.gov NCT05803460; https://clinicaltrials.gov/study/NCT05803460

## Introduction

### Population Aging and Mental Health Challenges

Population aging is a global phenomenon with profound implications for public health. As life expectancy continues to increase worldwide, a growing proportion of older adults experience age-related declines in cognitive functioning and emotional well-being [1,2]. Even among community-dwelling older adults who do not meet diagnostic criteria for cognitive impairment, subtle reductions in attention and mood are common and may precede more pronounced cognitive decline [3,4]. Consequently, identifying accessible and nonpharmacological strategies to support cognitive and emotional well-being has become a key priority for promoting healthy aging [5]. Although aging-related challenges are shared globally, contextual factors, such as urbanization, living environments, and health care infrastructure, substantially shape older adults’ daily experiences [6], underscoring the need for feasible interventions that can be integrated into everyday living contexts [7].

### Attention Decline and Attention Restoration Theory

Attention is a fundamental cognitive process that supports goal-directed behavior, information processing, and safe engagement in daily activities [8]. Age-related changes in attentional capacity, particularly those involving frontal brain networks, may compromise older adults’ ability to sustain attention and manage competing demands [9,10]. Because attention is essential for overall cognitive functioning, attentional impairments can adversely affect daily activities and social behavior, thereby increasing the risk of falls, unsafe driving, and accidental injuries [11,12]. Natural environments are recognized for providing restorative experiences that promote cognitive, psychological, and physiological well-being, particularly by facilitating attentional recovery [13,14]. Attention restoration theory (ART) provides a theoretical framework that explains how exposure to natural environments may facilitate attentional recovery [15,16]. According to ART, natural environments evoke “soft fascination,” allowing directed attention resources to rest and replenish [15]. Empirical studies have shown that exposure to natural settings is associated with improved attentional performance; however, findings remain mixed, particularly in studies using brief exposure durations or heterogeneous attentional measures [16,17]. These inconsistencies underscore the need for carefully designed trials that specifically examine attentional outcomes in older populations.

### Mood, Stress, and Stress Reduction Theory

Mood, defined as an individual’s emotional state, is a core component of psychological well-being and daily functioning [18]. In addition to attentional changes, older adults frequently experience emotional stress, anxiety, and depressive symptoms, which can negatively affect quality of life and may interact with cognitive decline [19,20]. In a community-based health survey, 36.9% of the adults aged more than 65 years exhibited anxiety symptoms, and 12.1% exhibited depressive symptoms [21]. Stress reduction theory suggests that natural environments elicit an affective response that promotes emotional calm and physiological relaxation through the regulation of the autonomic nervous system [22]. Empirical evidence from systematic reviews shows that exposure to natural or simulated natural environments is associated with reduced psychological distress, increased positive affect, and enhanced mood states [23,24].

### Barriers to Real-Nature Exposure and Virtual Nature as an Alternative

Despite the potential benefits of real-world natural environments, many older adults face barriers to accessing nature, including physical limitations, mobility constraints, transportation difficulties, urban density, and public health restrictions [25,26]. These barriers have prompted an increasing interest in virtual-nature interventions, which use immersive technologies to simulate natural environments in accessible, indoor settings [23]. Virtual-nature interventions use immersive digital simulations to recreate natural environments and elicit restorative effects comparable to those of real-world nature [27]. Systematic reviews have demonstrated that virtual-nature exposure can improve attention, cognitive performance, mood, and stress regulation [27,28]. Emerging evidence further suggests that even a single session of simulated nature exposure, lasting between 6 and 20 minutes, may reduce stress and enhance positive emotions and attentional restoration [29-32]. However, most previous studies have focused on younger or middle-aged adults, and evidence regarding the effectiveness of virtual-nature interventions among older adults remains limited [27,28]. In addition, the immediate effects of a single-session virtual-nature exposure on attention, mood, and physiological indicators in older populations are not well established. Moreover, few randomized controlled trials have directly compared virtual nature, actual nature, and urban environments within the same experimental framework among community-dwelling older adults.

To address these gaps, this study aimed to examine the immediate effects of a single-session virtual-nature intervention on attention and mood among community-dwelling older adults in Taiwan. Specifically, we investigated whether virtual or actual-nature exposure would produce greater improvements than exposure to an urban environment. We hypothesized that a single session of virtual or actual-nature exposure would lead to immediate improvements in attention, mood, and physiological outcomes.

## Methods

### Study Design

This study used a randomized controlled trial with a 3-group, pre-post design to examine the immediate effects of environmental exposure. The trial was registered at ClinicalTrials.gov (NCT05803460). Participants were randomly allocated to 1 of the 3 conditions: virtual nature, actual nature, or actual urban. Each participant completed a single 20-minute exposure session. Outcome measures were collected at baseline and immediately after the intervention to evaluate changes in attention, mood, and physiological responses.

### Participants

Participants were recruited from 6 community care centers in Taipei, Taiwan, between April and October 2023. Eligible participants were aged 65 years or older, had intact global cognitive function as assessed by the Short Portable Mental Status Questionnaire (>8), and were able to communicate in Mandarin Chinese and complete study procedures. Individuals were excluded if they had a diagnosed psychiatric disorder or medical condition that could interfere with study outcomes, severe visual or hearing impairments that would impede participation, or active ocular or dermatologic infectious diseases. The required sample size was determined using G*Power statistical software. Given the repeated-measures design and the primary interest in detecting group-by-time interaction effects, a small-to-medium effect size (Cohen *f*=0.20) was assumed based on an *F* test for ANOVA, as no previous studies using a comparable virtual-nature intervention design were available [33]. The significance level was set at an α of .05, with statistical power of 0.90. On the basis of these parameters, a total sample size of 84 participants was considered sufficient to detect overall intervention effects. After accounting for an anticipated 20% attrition rate, the minimum required sample size was increased to 105 participants; ultimately, 120 participants were enrolled.

This randomized controlled trial used computer-generated block randomization with a block size of 6. The allocation sequence was generated by a research team member who was not involved in participant recruitment or assessment. As participants were enrolled, the corresponding sequence numbers were placed individually into opaque, sealed envelopes to ensure allocation concealment. A separate research team member opened each envelope sequentially to assign participants to 1 of the 3 groups: the virtual-nature group, the actual-nature group, or the actual-urban group, with 40 participants allocated to each group.

### Intervention

Participants assigned to the virtual-nature group viewed a 20-minute immersive video of a natural environment filmed by the research team. This duration was selected based on previous evidence indicating that 10 to 20 minutes of virtual-nature exposure can improve attention and emotional outcomes in older adults [29,31]. The 360° panoramic video was recorded in a natural park in Taipei, a location commonly visited by older adults for its safe walking paths. Filming was conducted under stable weather conditions to minimize visual distractions and ensure ecological validity. The footage was captured using an Insta360 ONE R dual-lens camera, edited using standard audiovisual software, and structured into two 10-minute segments. In line with previous virtual-nature protocols for older adults, no interactive features were included to maintain a passive, restorative experience [34]. The video was delivered using a lightweight head-mounted display (HTC VIVE Flow). Before the session, participants received brief instructions encouraging them to observe in a relaxed manner. Each participant remained seated throughout the 20-minute exposure. For hygiene and safety, all equipment was disinfected after use, and disposable virtual reality (VR) face pads were provided.

Participants in the actual-nature group completed a 20-minute real-world exposure session at the same natural park featured in the virtual intervention. Upon arrival, participants rested for 10 to 15 minutes to acclimate. They were then seated in a safe, shaded location and instructed to observe their surroundings in a calm and relaxed manner.

Participants in the actual-urban group underwent a 20-minute observation session in a nearby urban area selected for safety and accessibility. As with the other groups, participants rested for 10 to 15 minutes before beginning the session and were seated during the exposure period, observing the environment in a relaxed state.

### Outcomes

#### Overview

At baseline, participants completed a questionnaire assessing demographic characteristics (age, sex, years of education, and marital status); health-related variables (BMI, blood pressure, exercise habits, sleep quality, and comorbidities); and time spent in natural environments. Additionally, perceived restorativeness was assessed after the intervention using the Perceived Restorativeness Scale [35]. The primary outcomes were attention and mood. The secondary outcomes were physiological indicators of stress.

#### Attention

Attention was assessed using the digit span test (DS) and the trail making test (TMT), which capture multiple dimensions of attentional functioning, including attentional span, orienting, and sustained attention. The DS includes 2 subtests: digit span forward (DSF) and digit span backward (DSB). In both tasks, participants were asked to immediately repeat digit sequences read aloud by the assessor, with testing discontinued after 2 consecutive errors. A total DS score was calculated by summing DSF (range 0-16) and DSB (range 0-14), with higher scores indicating better attentional capacity. The Chinese versions of DSF and DSB demonstrate acceptable sensitivity (0.62 and 0.77) and specificity (0.65 and 0.78), respectively [36]. The TMT assessed orienting and sustained attention and consisted of 2 parts. In part A, participants connected numbered circles (1-25) in ascending order, and in part B, they alternated between numbers (1-13) and letters. Performance was scored based on completion time, with longer times indicating poorer attention. The TMT has demonstrated strong reliability and validity as a measure of attentional processes [37].

#### Mood

Mood was assessed using the Positive and Negative Affect Schedule (PANAS), which evaluates 2 dimensions of affective states: positive affect (eg, enthusiasm and alertness) and negative affect (eg, distress and fear). The PANAS consists of 20 items, each rated on a 5-point Likert scale ranging from 1 (“very slightly or not at all”) to 5 (“extremely”). Higher scores indicated greater intensity of the respective affective states. The PANAS has demonstrated high internal consistency, with Cronbach α coefficients ranging from 0.86 to 0.90 for positive affect and 0.84 to 0.87 for negative affect [38].

#### Physiological Indicators of Stress

Physiological parameters were assessed using a wrist-worn autonomic monitoring device (ANSWatch, Taiwan Scientific Corporation; approved by the Taiwan Ministry of Health and Welfare, medical device permit number 001525). The device measured heart rate (HR), systolic blood pressure (SBP), diastolic blood pressure (DBP), and indices of sympathetic nervous system and parasympathetic nervous system activity. HR variability (HRV) indices were derived from normal-to-normal RR intervals. High-frequency (HF) power was used as an indicator of parasympathetic activity, whereas low-frequency (LF) power reflects combined autonomic influences. The LF/HF ratio was reported as an index of autonomic balance [31]. To reduce potential confounding, participants were instructed to avoid stimulant beverages (eg, tea or coffee) for at least 2 hours before measurement. All assessments were conducted while participants were seated comfortably in a relaxed position. Each HRV measurement required approximately 6 minutes.

### Procedure

Following approval from the institutional review board, community-dwelling older adults were recruited from 6 community health stations in Taipei City from April 2023 to October 2023. Station staff first identified and approached potential participants. Individuals who expressed interest were contacted by the research team for eligibility screening. After written informed consent was obtained, baseline assessments were conducted, including attention tests, mood questionnaires, and physiological measurements (HR, SBP, DBP, and HRV).

Participants were then exposed to 1 of the 3 assigned conditions (virtual nature, actual nature, or actual-urban environment) according to the randomized allocation sequence. A trained research assistant provided standardized instructions and supervised the 20-minute exposure session. Immediately after the intervention, participants completed the posttest assessments, which included physiological measurements, attention tests, and questionnaires. Participants were monitored for discomfort before the posttest assessment was initiated. The entire procedure required approximately 60 minutes per participant. All data were collected by a trained research assistant to ensure standardized administration of assessments and measurement reliability.

### Ethical Considerations

The study protocol was reviewed and approved by the institutional review board at National Yang Ming Chiao Tung University (YM111067). Written informed consent was obtained from all participants after they received a full explanation of the study purpose, procedures, potential risks, and expected benefits. Participants were informed that their involvement was voluntary and that they could withdraw from the study at any time without consequence. All members of the research team completed the required human participant protection training and certification before data collection began. A small gift was provided as appreciation for participation.

### Data Analysis

Data were analyzed using SPSS (version 25.0; IBM Corp). Descriptive statistics (means, SDs, frequencies, and percentages) were used to summarize participant characteristics and baseline variables. Intervention effects over time were analyzed using generalized estimating equations (GEEs) with an exchangeable working correlation structure and robust SEs. Group-by-time interaction effects were examined. In accordance with the CONSORT (Consolidated Standards of Reporting Trials) 2010 extension for multiarm randomized controlled trials (item 12a) [39], exploratory pairwise comparisons between each of the 2 intervention groups (virtual nature and actual nature) and the control group (urban environment) were conducted without adjustment for multiple comparisons. Baseline group differences and postintervention perceived restorativeness were examined using One-way ANOVA for continuous variables and the chi-square test for categorical variables. One-way ANOVA revealed a significant difference in postintervention perceived restorativeness scores among the 3 groups (*P*<.05). Post hoc analyses using the Tukey honestly significant difference test indicated significant between-group differences. A 2-tailed *P* value <.05 was considered statistically significant.

## Results

### Overview

**Figure 1:**
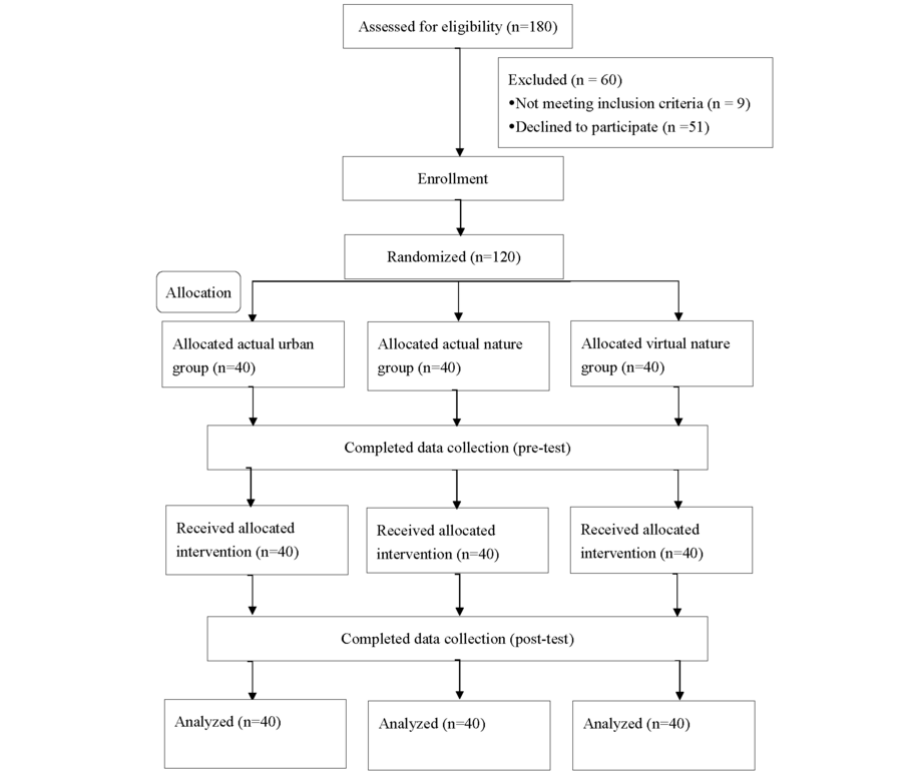
Flow diagram of the study.

A total of 180 invitation letters were distributed to community-dwelling older adults. Of these, 9 (5%) individuals did not meet the inclusion criteria, and 51 (28.3%) declined to participate. A total of 120 (66.7%) older adults agreed to participate, completed the study procedures, and were included in the final analysis (Figure 1). No adverse events or intervention-related discomfort was reported during the study.

### Sample Characteristics

A total of 120 community-dwelling older adults participated in this study. The mean age was 72.6 (SD 4.1) years, and most were female (n=97, 80.8%). Participants had an average of 10.2 (SD 2.8) years of formal education, and 79 (65.8%) were married. The mean BMI was 23.1 (SD 3.4) kg/m^2^. Most participants reported regular exercise (n=103, 85.8%), and more than half (n=70, 58.3%) perceived their sleep quality as poor. The most common chronic conditions were hypertension (n=49, 40.8%), hyperlipidemia (n=50, 41.7%), and diabetes (n=33, 27.5%). Regarding exposure to natural environments, 83 (69.2%) participants reported spending less than 1 hour per month in natural settings. Group comparisons showed no significant baseline differences in demographic or health-related characteristics, including age, sex, education, marital status, BMI, blood pressure, exercise habits, number of chronic conditions, or monthly nature exposure. However, a significant between-group difference was observed in perceived restorativeness. Post hoc analyses indicated that the virtual-nature group (*P*=.01) and the actual-nature group (*P*=.001) both reported significantly higher restorativeness scores than the actual-urban group (Table 1).

**Table 1:** Participants’ characteristics (N=120).

Characteristic		Total (N=120)	Virtual nature (n=40)	Actual nature (n=40)	Actual urban (n=40)	*P* value	
**Demographic variables**							
	Age (y), mean (SD)	72.6 (4.1)	72.2 (3.8)	72.0 (3.8)	73.5 (4.4)	.22	
	Female, n (%)	97 (80.8)	34 (85.0)	32 (80)	31 (77.5)	.69	
	Education (y), mean (SD)	10.2 (2.8)	10.6 (2.5)	9.9 (2.9)	10.1 (3)	.52	
**Marital status, n (%)**							.77
	Married	79 (65.8)	28 (70)	26 (65)	25 (62.5)		
	Single, divorced, or separated	41 (34.2)	12 (30)	14 (35)	15 (37.5)		
**Health-related variables, mean (SD)**							
	BMI (kg/m^2^)	23.1 (3.4)	23.5 (3.4)	22.8 (3.2)	23.1 (3.6)	.67	
	Systolic blood pressure	131.2 (16.9)	130.2 (17.9)	131.3 (14.2)	132.1 (18.5)	.89	
	Diastolic blood pressure	84.8 (9.6)	83.5 (8.8)	86.7 (9.8)	84.2 (10)	.30	
Having an exercise habit, n (%)		103 (85.8)	34 (85)	32 (80)	37 (92.5)	.27	
**Sleep quality, n (%)**							.97
	Good	50 (41.7)	17 (42.5)	16 (40)	17 (42.5)		
	Poor	70 (58.3)	23 (57.5)	24 (60)	23 (57.5)		
Number of comorbidities, mean (SD)		1.4 (1.1)	1.2 (0.9)	1.6 (1.2)	1.4 (1.1)	.28	
Hypertension, n (%)		49 (40.8)	15 (37.5)	14 (35)	20 (50)	.34	
Diabetes, n (%)		33 (27.5)	11 (27.5)	9 (22.5)	13 (32.5)	.61	
Hyperlipidemia, n (%)		50 (41.7)	14 (35)	16 (40)	20 (50)	.38	
**Nature-related variables: exposure to nature (monthly), n (%)**							.48
	≤1 h	83 (69.2)	30 (75)	25 (62.5)	28 (70)		
	>1 h	37 (30.8)	10 (25)	15 (37.5)	12 (30)		
Perceived restorativeness after intervention		19.8 (3.1)	20.3 (2.9)	20.8 (2.6)	18.3 (3.3)	.001^a^	

^a^Post hoc analysis of between-group comparisons using the Tukey honestly significant difference method: the virtual natural group>actual-urban group (*P*=.01); actual-nature group>actual-urban group (*P*=.001).

### Attention

GEE analyses revealed a significant overall group-by-time interaction for total DS scores (Wald *χ*^2^_2_=6.28; *P*=.04). Specifically, the pre-post improvement in attentional capacity was significantly greater in the virtual-nature group than in the actual-urban group (B=1.00, 95% CI 0.19-1.80; *P*=.02), indicating a greater increase in DS performance following exposure to virtual nature. The corresponding contrast between the actual-nature and actual-urban groups was not statistically significant. No significant overall group-by-time interaction effects were observed for performance on the TMT Part A (Wald *χ*^2^_2_=2.07; *P*=.36) or TMT Part B (Wald *χ*^2^_2_=1.90; *P*=.39) between the virtual-nature and actual-urban groups. Additionally, neither the virtual-nature group nor the actual-nature group showed significant improvements in attentional performance compared with the actual-urban group following the intervention (Table 2).

**Table 2:** Group means and effects of interaction for attention and mood (N=120).

Variables and group			Overall group×time Wald *χ*^2^ (*df*)^a^		Overall group×time *P* value		Before test, mean (SD)		After test, mean (SD)		B (95% CI)		Wald *χ*^2^ (*df*)		*P* value
**Digit span test**															
	Virtual nature	6.29 (2)		.04		24.4 (3.7)		25.8 (4.0)		1.00 (0.19 to 1.80)		5.88 (1)		.02	
	Actual nature	—^b^		—		25.4 (3.4)		26.5 (3.1)		0.57 (–0.03 to 0.93)		3.30 (1)		.12	
	Actual urban	—		—		25.6 (3.6)		26.0 (3.4)		—		—		—	
**TMT^c^ part A**															
	Virtual nature	2.07 (2)		.36		40.8 (20.7)		37.8 (15.4)		1.85 (–2.01 to 5.71)		0.87 (1)		.35	
	Actual nature	—		—		33.1 (15.6)		30.7 (12.4)		2.35 (–0.91 to 5.61)		1.98 (1)		.16	
	Actual urban	—		—		39.9 (16.7)		35.1 (14.8)		—		—		—	
**TMT part B**															
	Virtual nature	1.90 (2)		.39		83.9 (47.3)		65.9 (30.2)		–4.17 (–13.30 to 4.95)		0.80 (1)		.37	
	Actual nature	—		—		68.8 (34.3)		57.3 (27.2)		2.42 (–5.13 to 9.98)		0.39 (1)		.53	
	Actual urban	—		—		79.4 (46.3)		65.5 (37.1)		—		—		—	
**PANAS^d^ (positive)**															
	Virtual nature	11.46 (2)		.003		35.3 (6.8)		35.6 (7.5)		2.82 (0.02 to 5.62)		3.91 (1)		.048	
	Actual nature	—		—		33.1 (7.6)		34.2 (7.8)		3.92 (1.65 to 6.19)		11.46 (1)		.001	
	Actual urban	—		—		33.9 (6.9)		31.5 (8.4)		—		—		—	
**PANAS (negative)**															
	Virtual nature	6.05 (2)		.049		13.9 (4.0)		11.6 (2.8)		–2.22 (–4.05 to –0.39)		2.87 (1)		.02	
	Actual nature	—		—		14.5 (5.8)		12.7 (4.2)		–1.67 (–3.60 to 0.26)		2.87 (1)		.09	
	Actual urban	—		—		13.7 (4.4)		13.6 (5.7)		—		—		—	

^a^The generalized estimating equation analysis was conducted using the actual-urban group as the reference group; an omnibus Wald *χ*^2^_2_ test was first conducted for each outcome to evaluate the overall group-by-time interaction. Wald *χ*^2^_1_ tests were subsequently used to estimate individual group-by-time contrasts (virtual nature vs actual urban and actual nature vs actual urban).

^b^Not applicable.

^c^TMT: trail making test.

^d^PANAS: Positive and Negative Affect Schedule.

### Mood

Significant overall group-by-time interaction effects were observed for both positive affect (Wald *χ*^2^_2_=11.46; *P*=.003) and negative affect (Wald *χ*^2^_2_=6.05; *P*=.049). Compared with the urban environment group, the virtual-nature group demonstrated significantly greater improvements in both positive affect (B=2.82, 95% CI 0.02-5.62; *P*=.048) and negative affect (B=–2.22, 95% CI –4.05 to –0.39; *P*=.02). These findings indicate that exposure to virtual nature produced more pronounced mood-enhancing effects than the urban condition. In addition, the actual-nature group showed a significantly greater improvement in positive affect compared with the urban environment group (B=3.92, 95% CI 1.65-6.19; *P*=.001), whereas changes in negative affect did not reach statistical significance.

### Physiological Variables

GEEs analyses indicated that no significant overall group-by-time interaction effects were observed for any physiological indicators, including HR, SBP, DBP, LF, HF, or the LF/HF ratio (all omnibus Wald *χ*^2^_2_ tests; *P*>.05). Consistent with these findings, pairwise group-by-time contrasts showed no significant differences in pre-post changes between the virtual-nature and actual-urban groups or between the actual-nature and actual-urban groups (all Wald *χ*^2^_1_ tests; *P*>.05; Table 3).

**Table 3:** Group means and effects of interaction for physiological indicators of stress (N=120).

Variables and group		Overall group×time Wald *χ*^2^ (*df*)^a^	Overall group×time *P* value	Before test mean (SD)	After the test, mean (SD)				B (95% CI)		Wald χ^2^ (*df*)		*P* value
**Heart rate**													
	Virtual nature	3.29 (2)	.19	77.2 (10.5)	73.2 (8.8)				–1.23 (–3.38 to 0.92)		1.24 (1)		.26
	Actual nature	—^b^	—	77.1 (9.7)	75.6 (9.2)				1.17 (–1.31 to 3.67)		0.86 (1)		.36
	Actual urban	—	—	77.9 (9.4)	75.6 (9.0)				—		—		—
**Systolic blood pressure**													
	Virtual nature	2.82 (2)	.24	130.2 (17.9)	125.6 (16.0)				–0.97 (–8.46 to 6.50)		0.06 (1)		.80
	Actual nature	—	—	131.3 (14.2)	132.5 (17.9)				4.92 (–3.29 to 13.14)		1.37 (1)		.24
	Actual urban	—	—	132.1 (18.5)	128.2 (19.4)				—		—		—
**Diastolic blood pressure**													
	Virtual nature	0.57 (2)	.75	83.6 (8.8)	81.6 (8.5)				0.20 (–3.25 to 3.65)		0.01 (1)		.91
	Actual nature	—	—	86.7 (9.8)	85.6 (8.8)				1.31 (–2.21 to 4.83)		0.52 (1)		.47
	Actual urban	—	—	84.2 (10.0)	81.9 (9.4)				—		—		—
**LF^c^**													
	Virtual nature	1.43 (2)	.49	49.8 (20.7)	45.6 (16.6)			–4.76 (–13.06 to 3.52)		1.26 (1)		.26	
	Actual nature	—	—	46.0 (17.8)	45.5 (18.6)			–0.90 (–9.49 to 7.69)		0.04 (1)		.84	
	Actual urban	—	—	45.3 (17.4)	45.6 (19.4)			—		—		—	
**HF^d^**													
	Virtual nature	1.58 (2)	.45	50.1 (20.7)	54.4 (16.6)		4.75 (–3.53 to 13.05)			1.26 (1)		.26	
	Actual nature	—	—	54.9 (17.8)	53.8 (18.6)		0.21 (–8.56 to 8.99)			0.002 (1)		.96	
	Actual urban	—	—	54.6 (17.4)	54.3 (19.4)		—			—		—	
**LF/HF**													
	Virtual nature	4.42 (2)	.11	1.4 (1.1)	1.0 (0.7)	–0.45 (–0.93 to 0.03)					3.35 (1)		.07
	Actual nature	—	—	1.0 (0.7)	1.1 (0.9)	–0.03 (–0.52 to 0.45)					0.02 (1)		.89
	Actual urban	—	—	1.0 (0.8)	1.1 (0.9)	—					—		—

^a^The generalized estimating equation test was conducted using the actual-urban group as a reference; an omnibus Wald *χ*^2^_2_ test was first conducted for each outcome to evaluate the overall group-by-time interaction. Wald *χ*^2^_1_ tests were subsequently used to estimate individual group-by-time contrasts (virtual nature vs actual urban and actual nature vs actual urban).

^b^Not applicable.

^b^LF: low-frequency power.

^d^HF: high-frequency power.

## Discussion

### Principal Findings

This randomized controlled trial examined the immediate effects of a single-session virtual-nature exposure on attention, mood, and physiological indicators among community-dwelling older adults. The preliminary findings suggest that, compared to an urban environment, a single 20-minute exposure to virtual nature is associated with greater short-term improvements in attentional capacity and mood. Actual-nature exposure was associated with improved positive affect but did not produce significant attentional benefits. No significant physiological changes were observed across groups. These findings suggest that virtual nature may provide immediate cognitive and emotional benefits in older adults, particularly when access to real-world nature is limited. However, the observed effects were task and domain specific and should be interpreted cautiously within the context of a brief, single-session intervention.

Participants in the virtual-nature group demonstrated a statistically significant improvement in attentional span compared with those in the urban group. This result differed from several previous studies that reported nonsignificant effects of virtual-nature exposure on attention [31,40]. Differences in study design, attention assessments, sample characteristics, intervention duration, and limited statistical power may explain these inconsistent findings. Previous studies often relied on a single attention measure, such as the Sustained Attention to Response Test, which may not adequately capture the multifaceted nature of attention across environmental contexts [31]. Because distinct environments may engage different attentional components, the use of a single tool may limit sensitivity to change. This study used a multidimensional assessment approach, including the DS and TMT, to evaluate selective attention, sustained attention, and attentional span. This comprehensive measurement strategy may have enhanced the ability to detect subtle yet meaningful changes in attention following environmental exposure [14].

Improvements in attention were observed only in the DS and not in the TMT. This task-specific pattern may reflect differences in cognitive demands and sensitivity to short-term restorative effects. The DS primarily assesses attentional capacity and working memory [36], which may be more responsive to brief environmental restoration. In contrast, the TMT involves additional components, such as visual scanning, psychomotor speed, and executive control [37], which may require longer or repeated interventions to demonstrate measurable change. It is also noteworthy that the virtual-nature group had slightly lower baseline DS scores than the other groups, raising the possibility of a ceiling effect in the comparison groups [41]. Although the analyses focused on group-by-time interaction effects, baseline differences in performance may have influenced responsiveness to the intervention. Practice effects may also contribute to the absence of significant between-group differences in TMT performance [42]. Together, these findings highlight the importance of considering task characteristics and baseline performance when interpreting attention outcomes in environmental intervention studies.

Another explanation for the divergent findings may relate to differences in intervention duration and participant characteristics. This study used a single 20-minute session, whereas previous studies typically administered shorter 10-minute exposures in middle-aged and older adults [31] and younger adult samples [43]. Variations in exposure duration may influence the extent of attentional restoration achieved. In addition, because participants in this study were all aged 65 years or older, they may have been more responsive to restorative stimulation, as age-related declines in attentional capacity could increase susceptibility to the benefits of natural environments [44].

Both virtual and actual-nature exposure were associated with improvements in positive affect, with virtual nature additionally demonstrating reductions in negative affect. These results are consistent with previous research involving mixed middle-aged and older populations [31,45] and extend existing evidence by demonstrating similar effects in community-dwelling adults aged 65 years and older. These findings are broadly consistent with stress reduction theory, which posits that natural environments elicit affective responses that promote emotional calm and psychological well-being [22]. The emotional benefits observed following a single session suggest that even brief exposure to nature-related stimuli may have a positive influence on mood in older adults. The concurrent improvement in attention and mood in the virtual-nature group also aligns with ART [15,16], as emotional relaxation may facilitate the recovery of attentional resources. However, the absence of attentional benefits in the actual-nature group suggests that the relationship between environmental exposure and cognitive outcomes may depend on contextual factors, such as environmental familiarity, attentional engagement, and individual expectations.

A unique strength of this study was the use of locally familiar natural settings, which may have enhanced both the ecological relevance and acceptability of the intervention. Although most participants had no previous experience with VR, previous research suggests that Taiwanese older adults generally perceive VR as useful, enjoyable, and easy to use for supporting active aging [46]. Such positive attitudes may have facilitated greater immersion and engagement with the virtual natural environment.

Contrary to the initial hypothesis, no significant changes in physiological indicators of stress, including HRV and blood pressure, were observed over time between the virtual-nature and urban groups or between the actual-nature and urban groups. Thus, the findings are consistent with previous research reporting similar null effects [31,45]. Several factors may account for these results. First, the single 20-minute intervention may have been insufficient to elicit detectable changes in the autonomic nervous system, particularly in older adults. Second, most participants (102/120, 85%) had no previous experience with VR, and novelty-induced arousal associated with first-time VR exposure may have transiently increased sympathetic activation [47,48], potentially masking relaxation-related physiological responses.

### Limitations

Several limitations should be acknowledged. First, the sample comprised predominantly female, community-dwelling older adults recruited from urban areas in northern Taiwan. This demographic profile may limit the generalizability of the findings to male older adults, individuals living in rural settings, those with mobility limitations or limited familiarity with technology, and older adults from different cultural contexts. Future research should explicitly examine the feasibility and effectiveness of the intervention in more diverse and vulnerable older populations. Second, this study was designed as a preliminary, exploratory investigation of immediate effects. Guided by feasibility considerations and real-world conditions, this study used a single-session intervention design and included an urban environment as a comparison group. Future research is recommended to conduct larger-scale trials with multiple intervention sessions and to incorporate additional no-exposure control conditions to further validate the findings. Third, most (102/120, 85%) participants in this study had no previous experience with VR. As a result, the observed effects may partially reflect VR-related factors, such as novelty or immersion, rather than the natural content alone, particularly with respect to physiological outcomes (eg, HRV). In addition, this study included only a single VR condition and did not incorporate formal measures of presence or engagement, which limits the interpretation of the underlying mechanisms of the observed effects. Future research should use multiple VR control conditions and incorporate validated measures of presence or immersion to strengthen the evidential support for the validity of the intervention. Finally, the study sample consisted of older adults who were able to participate in community activities, excluding those with limited mobility. Future studies should include mobility-impaired older adults to further evaluate the feasibility, accessibility, and effectiveness of virtual-nature interventions in broader older populations.

### Conclusions

The findings of this study provide evidence that a single 20-minute virtual-nature exposure can yield immediate improvements in attention and mood among community-dwelling older adults, whereas physiological effects were not detected. Virtual nature may represent a feasible and accessible short-term restorative strategy, particularly for older adults facing barriers to accessing real-world natural environments. Future research using multisession interventions, larger and more diverse samples, additional control conditions, and longitudinal follow-up is needed to clarify sustained effects and underlying mechanisms.

## Appendix

Multimedia Appendix 1 CONSORT-EHEALTH checklist (V 1.6.1).

## Abbreviations

ART: attention restoration theory
CONSORT: Consolidated Standards of Reporting Trials
DBP: diastolic blood pressure
DS: digit span test
DSB: digit span backward
DSF: digit span forward
GEE: generalized estimating equation
HF: high frequency
HR: heart rate
HRV: heart rate variability
LF: low frequency
PANAS: Positive and Negative Affect Schedule
SBP: systolic blood pressure
TMT: trail making test
VR: virtual reality

## Data Availability

The datasets generated or analyzed during this study are available from the corresponding author on reasonable request.
