# Supplementary material for: Effects of a Virtual Reality–Based Natural Environment Intervention on Attention and Mood in Community-Dwelling Older Adults: Randomized Controlled Trial
Source: JMIR Aging. 2026 Feb 25;9:e87861. doi: 10.2196/87861 (PMC12980055; doi:10.2196/87861)
Supplement: Multimedia Appendix 1 [file aging_v9i1e87861_app1.pdf]

CONSORT-EHEALTH Checklist V1.6.2 Report	Manuscript Number	87861
**(based on CONSORT-EHEALTH V1.6), available at [http://tinyurl.com/consort-ehealth-v1-6].**		
**Date completed**		
2/14/2026 4:24:24		
**by**		
Cheng-Chen Chou		
TITLE		
**1a-i) Identify the mode of delivery in the title**		
**1a-ii) Non-web-based components or important co-interventions in title**		
Mode of delivery is stated at the beginning of the title: "a Virtual Reality–Based Natural Environment Intervention'		
**1a-iii) Primary condition or target group in the title**		
ABSTRACT		
**1b-i) Key features/functionalities/components of the intervention and comparator in the METHODS section of the ABSTRACT**		
**1b-ii) Level of human involvement in the METHODS section of the ABSTRACT**		
As described in the Methods section, participants were randomly allocated to one of three groups: the virtual nature group, the actual nature group, or the urban environment group.		
**1b-iii) Open vs. closed, web-based (self-assessment) vs. face-to-face assessments in the METHODS section of the ABSTRACT**		
**1b-iv) RESULTS section in abstract must contain use data**		
**1b-v) CONCLUSIONS/DISCUSSION in abstract for negative trials**		
This is clearly addressed in the abstract: " pre-post design was conducted among 120 community-dwelling adults aged 65 years and older in northern Taiwan. Participants were randomly assigned to 1 of the 3 groups."		
INTRODUCTION		
**2a-i) Problem and the type of system/solution**		
**2a-ii) Scientific background, rationale: What is known about the (type of) system**		
Even among community-dwelling older adults who do not meet diagnostic criteria for cognitive impairment, subtle reductions in attention and mood are common and may precede more pronounced cognitive decline. Consequently, identifying accessible and nonpharmacological strategies to support cognitive and emotional well-being has become a key priority for promoting healthy aging. Although aging-related challenges are shared globally, contextual factors, such as urbanization, living environments, and health care infrastructure, substantially shape older adults' daily experiences, underscoring the need for feasible interventions that can be integrated into everyday living contexts		
METHODS		
**3a) CONSORT: Description of trial design (such as parallel, factorial) including allocation ratio**		
**3b) CONSORT: Important changes to methods after trial commencement (such as eligibility criteria), with reasons**		
we investigated whether virtual or actual nature exposure would produce greater improvements than exposure to an urban environment. We hypothesized that a single session of virtual or actual nature exposure would lead to immediate improvements in attention, mood, and physiological outcomes.		
**3b-i) Bug fixes, Downtimes, Content Changes**		
**4a) CONSORT: Eligibility criteria for participants**		
This study used a randomized controlled trial with a 3-group, pre-post design to examine the immediate effects of environmental exposure. The trial was registered at ClinicalTrials.gov (NCT05803460). Participants were randomly allocated to 1 of the 3 conditions: virtual nature, actual nature, or actual urban environment. Each participant completed a single 20-minute exposure session. Participants were recruited from 6 community care centers in Taipei.		
**4a-i) Computer / Internet literacy**		
**4a-ii) Open vs. closed, web-based vs. face-to-face assessments:**		
**4a-iii) Information giving during recruitment**		
Station staff first identified and approached potential participants. Individuals who expressed interest were contacted by the research team for eligibility screening. After written informed consent was obtained, baseline assessments were conducted, including attention tests, mood questionnaires, and physiological measurements. Participants were then exposed to 1 of the 3 assigned conditions (virtual nature, actual nature, or actual urban environment) according to the randomized allocation sequence. A trained research assistant provided standardized instructions and supervised the 20-minute exposure session. Immediately after the intervention, participants completed the posttest assessments, which included physiological measurements, attention tests, and questionnaires. Participants were monitored for discomfort before the posttest assessment was initiated. The entire procedure required approximately 60 minutes per participant. All data were collected by a trained research assistant to ensure standardized administration of assessments and measurement reliability.		
**4b) CONSORT: Settings and locations where the data were collected**		
not applicable; there were no changes to methods after trial commencement		
**4b-i) Report if outcomes were (self-)assessed through online questionnaires**		
**4b-ii) Report how institutional affiliations are displayed**		
This is not applicable. Online questionnaires were not used with this study.		
**5) CONSORT: Describe the interventions for each group with sufficient details to allow replication, including how and when they were actually administered**		
**5-i) Mention names, credential, affiliations of the developers, sponsors, and owners**		
**5-ii) Describe the history/development process**		
**5-iii) Revisions and updating**		
**5-iv) Quality assurance methods**		
**5-v) Ensure replicability by publishing the source code, and/or providing screenshots/screen-capture video, and/or providing flowcharts of the algorithms used**		
**5-vi) Digital preservation**		
**5-vii) Access**		
**5-viii) Mode of delivery, features/functionalities/components of the intervention and comparator, and the theoretical framework**		

Participants assigned to the virtual nature group viewed a 20-minute immersive video of a natural environment filmed by the research team. The video was delivered using a lightweight head-mounted display (HTC VIVE Flow). Each participant remained seated throughout the 20-minute exposure. For hygiene and safety, all equipment was disinfected after use, and disposable virtual reality (VR) face pads were provided.<br>Participants in the actual nature group completed a 20-minute real-world exposure session at the same natural park featured in the virtual intervention.<br>Participants in the actual urban group underwent a 20-minute observation session in a nearby urban area selected for safety and accessibility.		
**5-ix) Describe use parameters**		
Participants assigned to the virtual nature group viewed a 20-minute immersive video of a natural environment filmed by the research team. This duration was selected based on previous evidence indicating that 10 to 20 minutes of virtual nature exposure can improve attention and emotional outcomes in older adults [29,31]. The 360° panoramic video was recorded in a natural park in Taipei, a location commonly visited by older adults for its safe walking paths. Filming was conducted under stable weather conditions to minimize visual distractions and ensure ecological validity. The footage was captured using an Insta360 ONE R dual-lens camera, edited using standard audiovisual software, and structured into two 10-minute segments. In line with previous virtual nature protocols for older adults, no interactive features were included to maintain a passive, restorative experience. The video was delivered using a lightweight head-mounted display (HTC VIVE Flow). Before the session, participants received brief instructions encouraging them to observe in a relaxed manner. Each participant remained seated throughout the 20-minute exposure. For hygiene and safety, all equipment was disinfected after use, and disposable virtual reality (VR) face pads were provided. Participants in the actual nature group completed a 20-minute real-world exposure session at the same natural park featured in the virtual intervention. Upon arrival, participants rested for 10 to 15 minutes to acclimate. They were then seated in a safe, shaded location and instructed to observe their surroundings in a calm and relaxed manner. Participants in the actual urban group underwent a 20-minute observation session in a nearby urban area selected for safety and accessibility. As with the other groups, participants rested for 10 to 15 minutes before beginning the session and were seated during the exposure period, observing the environment in a relaxed state.		
**5-x) Clarify the level of human involvement**		
**5-xi) Report any prompts/reminders used**		
**5-xii) Describe any co-interventions (incl. training/support)**		
This intervention did not have any prompts to remind participants to engage.		
**6a) CONSORT: Completely defined pre-specified primary and secondary outcome measures, including how and when they were assessed**		
Eligibility criteria for both participant groups were clearly outline" Eligible participants were aged 65 years or older, had intact global cognitive function as assessed by the Short Portable Mental Status Questionnaire (>8), and were able to communicate in Mandarin Chinese and complete study procedures. Individuals were excluded if they had a diagnosed psychiatric disorder or medical condition that could interfere with study outcomes, severe visual or hearing impairments that would impede participation, or active ocular or dermatologic infectious diseases."		
**6a-i) Online questionnaires: describe if they were validated for online use and apply CHERRIES items to describe how the questionnaires were designed/deployed**		
**6a-ii) Describe whether and how "use" (including intensity of use/dosage) was defined/measured/monitored**		
**6a-iii) Describe whether, how, and when qualitative feedback from participants was obtained**		
**6b) CONSORT: Any changes to trial outcomes after the trial commenced, with reasons**		
This study used a randomized controlled trial with a 3-group, pre-post design to examine the immediate effects of environmental exposure. Participants were recruited from 6 community care centers in Taipei, Taiwan.		
**7a) CONSORT: How sample size was determined**		
**7a-i) Describe whether and how expected attrition was taken into account when calculating the sample size**		
**7b) CONSORT: When applicable, explanation of any interim analyses and stopping guidelines**		
The primary outcomes were attention and mood. The secondary outcomes were physiological indicators of stress. Each participant completed a single 20-minute exposure session. Outcome measures were collected at baseline and immediately after the intervention to evaluate changes in attention, mood, and physiological responses.		
**8a) CONSORT: Method used to generate the random allocation sequence**		
There was no changes to trial outcomes after the trial commenced.		
**8b) CONSORT: Type of randomisation; details of any restriction (such as blocking and block size)**		
Not applicable, interim analyses were not included in this study design.		
**9) CONSORT: Mechanism used to implement the random allocation sequence (such as sequentially numbered containers), describing any steps taken to conceal the sequence until interventions were assigned**		
computer-generated method was used to generate the random allocation sequence.		
**10) CONSORT: Who generated the random allocation sequence, who enrolled participants, and who assigned participants to interventions**		
This is addressed in the Randomization subsection in the Methods section: "This randomized controlled trial used computer-generated block randomization with a block size of 6."		
**11a) CONSORT: Blinding - If done, who was blinded after assignment to interventions (for example, participants, care providers, those assessing outcomes) and how**		
**11a-i) Specify who was blinded, and who wasn't**		
**11a-ii) Discuss e.g., whether participants knew which intervention was the "intervention of interest" and which one was the "comparator"**		
This concern has been addressed in the Methods section. Allocation bias was minimized through allocation concealment, and outcome assessors were blinded to group assignment prior to baseline assessment.		
**11b) CONSORT: If relevant, description of the similarity of interventions**		
This is addressed in the Randomization subsection in the Methods section: " the corresponding sequence numbers were placed individually into opaque, sealed envelopes to ensure allocation concealment. A separate research team member opened each envelope sequentially to assign participants to 1 of the 3 groups."		
**12a) CONSORT: Statistical methods used to compare groups for primary and secondary outcomes**		
the full trial protocol can be accessed: https://clinicaltrials.gov/study/NCT05803460		
**12a-i) Imputation techniques to deal with attrition / missing values**		
**12b) CONSORT: Methods for additional analyses, such as subgroup analyses and adjusted analyses**		
This issue is addressed in detail in the Intervention Group subsections of the Methods section.		
RESULTS		
**13a) CONSORT: For each group, the numbers of participants who were randomly assigned, received intended treatment, and were analysed for the primary outcome**		
**13b) CONSORT: For each group, losses and exclusions after randomisation, together with reasons**		
This is addressed in the Methods section: "Baseline group differences and postintervention perceived restorativeness were examined using one-way ANOVA for continuous variables and the chi-square test for categorical variables."		
**13b-i) Attrition diagram**		
**14a) CONSORT: Dates defining the periods of recruitment and follow-up**		
This is addressed in the Overview subsection in the Results section: "A total of 180 invitation letters were distributed to community-dwelling older adults. Of these, 9 (5%) individuals did not meet the inclusion criteria, and 51 (28.3%) declined to participate. A total of 120 (66.7%) older adults agreed to participate, completed the study procedures, and were included in the final analysis."		
**14a-i) Indicate if critical "secular events" fell into the study period**		
**14b) CONSORT: Why the trial ended or was stopped (early)**		

Not applicable. There were no participants lost to follow-up.		
**15) CONSORT: A table showing baseline demographic and clinical characteristics for each group**		
This is addressed in Procedure subsection in the Methods section: "Following approval from the institutional review board, community-dwelling older adults were recruited from 6 community health stations in Taipei City from April 2023 to October 2023."		
**15-i) Report demographics associated with digital divide issues**		
**16a) CONSORT: For each group, number of participants (denominator) included in each analysis and whether the analysis was by original assigned groups**		
**16-i) Report multiple "denominators" and provide definitions**		
**16-ii) Primary analysis should be intent-to-treat**		
This information is presented in Tables 1–3 and their corresponding descriptions in the Results section, where the appropriate denominators for participants included in the primary and secondary outcome analyses are clearly specified.		
**17a) CONSORT: For each primary and secondary outcome, results for each group, and the estimated effect size and its precision (such as 95% confidence interval)**		
Not applicable, the trial was not stopped early.		
**17a-i) Presentation of process outcomes such as metrics of use and intensity of use**		
**17b) CONSORT: For binary outcomes, presentation of both absolute and relative effect sizes is recommended**		
This is addressed by Table 1 in Participants' characteristics subsection in Result section.		
**18) CONSORT: Results of any other analyses performed, including subgroup analyses and adjusted analyses, distinguishing pre-specified from exploratory**		
This information is presented in Tables 2–3. Specifically, the pre–post improvement in attentional capacity was significantly greater in the virtual nature group than in the actual urban group (B = 1.00, 95% CI 0.19–1.80; P = .02). No significant overall group-by-time interaction effects were observed for performance on the TMT Part A (Wald $\chi^2(2)$ = 2.07; P = .36) or TMT Part B (Wald $\chi^2(2)$ = 1.90; P = .39) between the virtual nature and actual urban groups. Compared with the urban environment group, the virtual nature group demonstrated significantly greater improvements in both positive affect (B = 2.82, 95% CI 0.02–5.62; P = .048) and negative affect (B = –2.22, 95% CI –4.05 to –0.39; P = .02). Consistent with these findings, pairwise group-by-time contrasts showed no significant differences in pre–post changes between the virtual nature and actual urban groups or between the actual nature and actual urban groups (all Wald $\chi^2(1)$ tests; P > .05; Table 3).		
**18-i) Subgroup analysis of comparing only users**		
**19) CONSORT: All important harms or unintended effects in each group**		
Not applicable, as no binary outcomes were included in this study.		
**19-i) Include privacy breaches, technical problems**		
**19-ii) Include qualitative feedback from participants or observations from staff/researchers**		
DISCUSSION		
**20) CONSORT: Trial limitations, addressing sources of potential bias, imprecision, multiplicity of analyses**		
**20-i) Typical limitations in ehealth trials**		
**21) CONSORT: Generalisability (external validity, applicability) of the trial findings**		
**21-i) Generalizability to other populations**		
**21-ii) Discuss if there were elements in the RCT that would be different in a routine application setting**		
**22) CONSORT: Interpretation consistent with results, balancing benefits and harms, and considering other relevant evidence**		
**22-i) Restate study questions and summarize the answers suggested by the data, starting with primary outcomes and process outcomes (use)**		
**22-ii) Highlight unanswered new questions, suggest future research**		
This is addressed in the Discussion section: "This randomized controlled trial examined the immediate effects of a single-session virtual nature exposure on attention, mood, and physiological indicators among community-dwelling older adults. The preliminary findings suggest that, compared to an urban environment, a single 20-minute exposure to virtual nature is associated with greater short-term improvements in attentional capacity and mood. Actual nature exposure was associated with improved positive affect but did not produce significant attentional benefits. No significant physiological changes were observed across groups. These findings suggest that virtual nature may provide immediate cognitive and emotional benefits in older adults, particularly when access to real-world nature is limited. However, the observed effects were task and domain specific and should be interpreted cautiously within the context of a brief, single-session intervention."		
Other information		
**23) CONSORT: Registration number and name of trial registry**		
Not applicable, as no additional analyses, including subgroup or adjusted analyses, were performed in this study.		
**24) CONSORT: Where the full trial protocol can be accessed, if available**		
Not applicable, as no harms or unintended effects were observed in either intervention arm at the study sites during the study period.		
**25) CONSORT: Sources of funding and other support (such as supply of drugs), role of funders**		
This is addressed in the Study Design subsection in the Methods section: "The trial was registered at ClinicalTrials.gov (NCT05803460)."		
**X26-i) Comment on ethics committee approval**		
**x26-ii) Outline informed consent procedures**		
**X26-iii) Safety and security procedures**		
**X27-i) State the relation of the study team towards the system being evaluated**		
